# Fragment-level FAIRness: annotating scientific data and its provenance using data fragment selectors

**DOI:** 10.1515/jib-2025-0052

**Published:** 2026-06-17

**Authors:** Heinrich Lukas Weil, Kevin Schneider, Dominik Brilhaus, Timo Mühlhaus, David Zimmer

**Affiliations:** RPTU Kaiserslautern-Landau, Faculty of Biology, Computational Systems Biology Department, D-67663 Kaiserslautern, Germany; Heinrich Heine University Düsseldorf, Faculty of Mathematics and Natural Sciences, Cluster of Excellence on Plant Sciences (CEPLAS), D-40225 Düsseldorf, Germany

**Keywords:** research data management, fragment selectors, datamap, ISA, RO-Crate, ARC

## Abstract

Scientific communication depends on the production of *FAIR* (findable, accessible, interoperable, reusable) data. Yet when datasets are shared, they remain hard to interpret across domains because annotations are tied to heterogeneous file formats and implicit semantics. We present a *format-agnostic* method for *fragment-level* annotation based on *Data Fragment Selectors (DFS)*: *URI*-addressable pointers to sub-file regions (e.g., CSV rows/columns, JSON objects) whose meaning is defined by an accompanying explicit selector standard. DFS are aggregated into a *Datamap*, a lightweight schema that binds referenced fragments to *ontology*-backed semantics, including units and object types. Separating structural addressing from semantic interpretation enables reusable schemas and robust automation without format-specific parsing. To support provenance annotation, we integrate DFS with the Investigation-Study-Assay (*ISA*) metadata model, extending data-entity references to enable *sub-file granularity*. We realize the model in two interoperable serializations: *ISA-XLSX* for spreadsheet-centric practice and an Research Object Crate *(RO-Crate)* Datamap profile for web-native packaging and exchange. These specifications deliver *fragment-level FAIRness*: precise, *machine-actionable* links between process metadata and the data portions they describe. We demonstrate practical applicability by introducing a *Spreadsheet Fragment Identification* specification, providing a standardized workflow for annotating aggregated supplementary data in journal publications, and via programmatic exploration of a real-world proteomics project.

## 1 Introduction

Scientific discovery increasingly depends on the sharing and reuse of research data. Data can generate insights far beyond the original study’s scope only when they are not merely accessible but understandable and interpretable by both humans and machines. The FAIR (Findable, Accessible, Interoperable, Reusable) principles have become the dominant guidelines for directing data stewardship [1]. However, achieving FAIR in practice remains challenging: recent analyses show a proliferation of FAIR-assessment tools and indicators that apply different checks and scoring strategies, producing inconsistent results across domains and repositories. This heterogeneity complicates interpretation of “FAIR” scores and undermines trust in automated assessments [2], 3]. A central driver of these inconsistencies is metadata and annotation quality. When variables lack clear definitions or provenance is incomplete, even publicly available datasets are difficult to reuse or reproduce [4].

The importance of thorough annotation becomes especially evident in the life sciences, where research data are generated in a wide array of heterogeneous formats. Established community standards exist for many types of biological data. For example, **FASTA** and **FASTQ** formats dominate sequence representation and are widely deposited in resources such as GenBank, EMBL-EBI’s European Nucleotide Archive (ENA), and NCBI’s Sequence Read Archive(SRA) [5], [6], [7], [8]. For proteomics, the **mzML** and **mzIdentML** formats provide standardized representations of mass spectrometry data, supported by repositories like PRIDE [9]. Structural biology relies on **PDB/mmCIF** formats for macromolecular structures, hosted by the Protein Data Bank [10]. Similarly, large-scale expression studies often use **GTF/GFF** for gene annotations, with data deposited in GEO or ArrayExpress [11], 12]. These file formats, together with the database schemas that underpin them, have played a crucial role in promoting interoperability and reuse across subfields of biology.

Yet, despite these successes, the velocity of experimental innovation in biology consistently outpaces the development of rigid file standards. Emerging high-throughput methods, multi-omics integration, and single-cell assays generate new data modalities faster than standardized formats can be agreed upon. As a result, researchers frequently resort to more flexible, lightweight representations such as **CSV** tables or **JSON** objects to capture complex, custom experimental outputs [13], 14]. These formats support rapid dissemination and can be easily parsed by modern computational pipelines, but they lack built-in semantics: column names may be ambiguous, hierarchies implicit, and metadata inconsistent across labs. Without additional annotation layers, such files are difficult to interpret, integrate, or validate beyond their original context.

This tension underscores the need for **schematization standards** that transcend individual file formats. Rather than prescribing a fixed representation for each data type, schematization frameworks define the semantic structure, relationships, and metadata requirements that any data must satisfy. Such approaches are already emerging in biology through schema languages (e.g., JSON Schema, LinkML) and community efforts to capture metadata models for diverse omics datasets [15], 16]. In this work, we want to propose an alternative route. Embedded into the general framework of the Annotated Research Context (ARC) [17], 18] and built on top of existing specifications and technologies described below, we present the Datamap and the integration of Data Fragment Selectors [19] with the ISA framework [20]. In this approach, we focus on decoupling semantic from structural data file annotation, maximizing both explicitness and genericity.

## 2 Related works

Recent scholarship has brought up various format-agnostic mechanisms for expressing the meaning of data. By decoupling semantics from concrete representations, these approaches provide a durable basis for interoperability and reuse as formats and modalities evolve. In practice, useful annotation spans several layers: (i) packaging of datasets as coherent research objects, (ii) contextualization and provenance of how data were produced, and (iii) file-segment-level descriptions of internal structure and semantics. This categorization is not meant as a strict definition, but will help us differentiate the tasks we want to solve with our work, and which approach we followed for which task.

(i) Definition of packaging structure:

Packaging of datasets as coherent research objects is a baseline requirement for the implementation of FAIR digital objects. This layer of annotation creates a common ground for the overall research object structure, containing both data and its accompanying metadata. To this effect, BagIt (RFC 8493) defines a robust, manifest-driven layout for moving and preserving digital content; importantly, it is intentionally agnostic to a payload’s internal semantics [21]. Frictionless Data Package [22] on the other hand adds a lightweight JSON descriptor for datasets that enumerates resources and basic metadata, and can be specialized (e.g., Tabular Data Package) for consistent distribution and tooling. Going further on machine-actionable semantics, RO-Crate provides a JSON-LD–based annotation file centered on typed objects and explicit links among them. The core specification defines only a handful of constraints, including file and folder annotation. It encourages use of a shared data model and vocabulary for general annotation, mostly based on Schema.org, but also enables domain-specific schematization for sub-communities through the use of profiles [23].

(ii) Contextualization and provenance:

The contextualization and provenance of how data were produced does not necessarily need to be specified in a specific format. Instead, this can be an abstract data model, ontology or even information checklist. One such information checklist is Minimal Information About a Plant Phenotyping Experiment (MIAPPE), defining the expected research entities and their connections, specific to the subdomain of plant phenotyping [24]. For more generic biological or even chemical experiments, the ISA framework (Investigation–Study–Assay) captures the research entities in a more generic approach [20]. Through parametrized processes, the various entities (either real-world or data), are associated into input/output relationships. This generic model can then be infused with domain-specificity through the use of ontology terms attached to the entities in the resulting process graph [20], 25]. One more domain specific approach is the Sample and Data Relationship Format in Proteomics (SDRF-Proteomics), which further constrains the given information towards proteomics experiments, fixating it in a full specification [26]. ISA and other provenance annotation models complement packaging by describing how data was aquired, information essential for correct reuse.

(iii) File-segment-level descriptions:

File-segment-level descriptions of internal structure and semantics refer to annotation that breaks the file boundary, guiding file parsing and aiding interpretation by assigning semantic value to the entities contained in the file. A very generic but verbose approach is LinkML: it is a YAML-based schematization language which is meant to help handling and validating data while aligning entities and slots with ontologies [16]. More specific to tabular data, Frictionless Table Schema specifies field types, constraints (e.g., unique, minimum, pattern), missing-value policies, and inter-table relations via *primaryKey* and *foreignKeys*, offering a pragmatic, JSON-native model for tabular data [22]. Both these approaches have in common, that the structure of the annotation file mimics that of the data file. In other words, internal structure and semantic annotation are closely linked. A possible separation of these tasks is enabled by selectors. The W3C Web Annotation Data Model defines selectors for binding annotations to specific sub-resources or text spans within a larger resource [27]. For tabular data specifically, RFC7111 standardizes URI fragment identifiers that address rows, columns, and individual cells, making it possible to cite or annotate precise table regions [19].

The ARC is a framework meant to encompass the whole research cycle [17]. One of the main goals is to follow existing standards, increase interoperability and lower the barriers of community adoption [28]. To this effect, it is a real-world implementation of an RO-Crate as a packaging container (i). Experimental context and provenance are annotated through the use of the ISA abstract model (ii), which has been represented in JSON-LD and integrated into the RO-Crate framework through the creation of an ISA-RO-Crate profile [29]. Here, we propose our solution for file-segment-level descriptions (iii) and showcase their concrete implementation with real-world examples.

## 3 Scientific data annotation using data fragment selectors

In this work, we propose a general-purpose and data-format-agnostic approach to scientific data and data provenance annotation, based on the concept of Data Fragment Selectors (DFSs), as defined by the W3C Web Annotation Data Model [27]. A DFS is a singular string encoding pointers to specific regions of files or other digital resources, originally part of the fragment part of URLs (which themselves are the last part of URLs, denoted with a *#*). These selectors can target individual cells, ranges of cells, lines, or byte ranges in a file.

**Figure 1:**
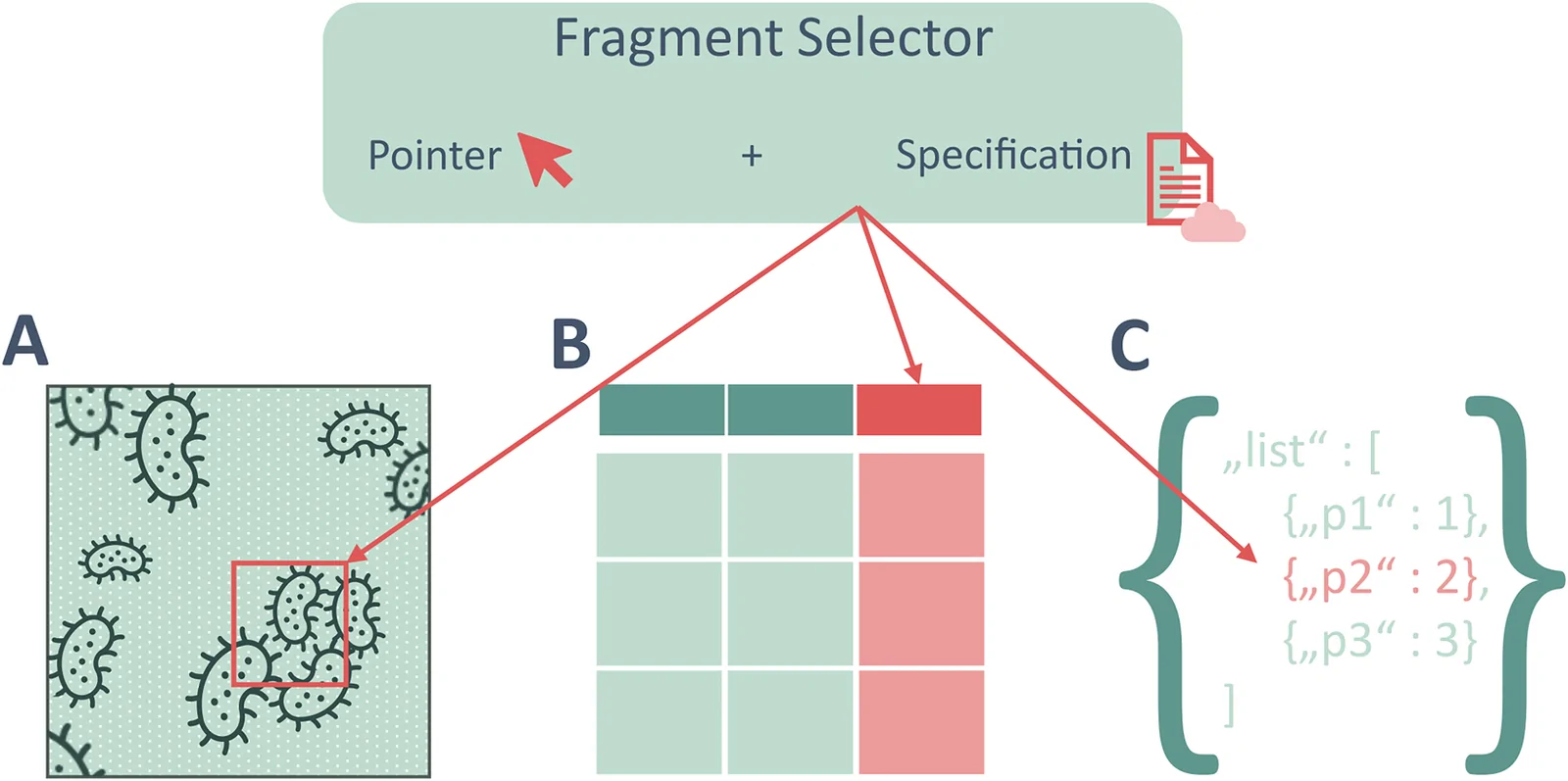
Coupled with an applicable selector specification, Fragment Selectors can point to – i.e. reference – segments in any file. Common use cases in biology include (A) areas of interest in image data, (B) columns in tabular data or (C) objects in JSON data.

A DFS consists of the *segment identifier* (or *pointer*) itself (e.g. *#xywh = 100, 200, 50, 50* or *#cell = R3C2*) and a reference to the interpretation standard that explains how that identifier should be parsed and understood (illustrated in Figure 1). Through this generalization, references to DFSs can be extended to any file format that has a defined method for referencing its internal structure.

### 3.1 Datamap

**Figure 2:**
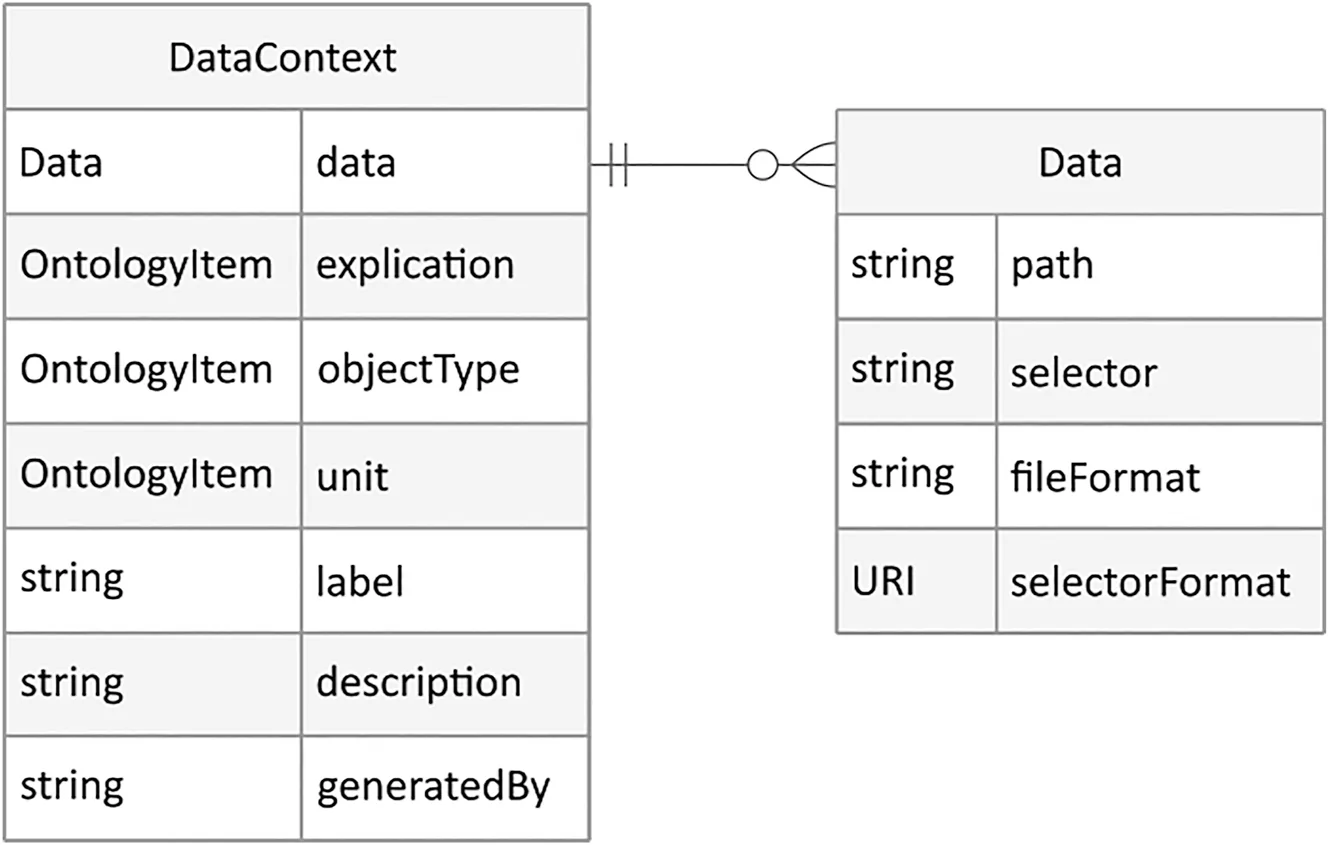
The data object in itself contains all information needed to locate the segment of interest. A Data Context just provides a thin layer of annotation around the data object, making use of ontologies where values are expected to follow a shared vocabulary.

We introduce the *Datamap* as a lightweight, file-format-agnostic annotation layer that attaches precise, machine-readable semantics to arbitrarily small fragments of research data. Conceptually, each entry of a Datamap is a *Data Context*, a compact record that binds a selected fragment of a file to its meaning and use. The file fragment is addressed by a data object, which encapsulates all access details: a path to the resource, an optional fragment selector (e.g., a column, range, region, or time slice), and the corresponding *File Format* and *Selector Format* that specify how the resource and selector are to be interpreted (Figure 2). All file-specific logic is therefore wrapped inside the data selector and its specification, while the surrounding annotation remains stable. This separation of concerns renders the Data Context structurally invariant across data formats, whether the fragment resides in a CSV column, a JSON object, or a binary stream, the same annotation schema applies.

Each Data Context provides a thin but expressive semantic layer around the data object. The field *Explication* states the conceptual meaning of the fragment (e.g., gene identifier, peak area), *Object Type* captures the expected data type or structure (e.g., string, integer, vector), and *Unit* records the measurement unit where applicable. *Label* preserves the local identifier used at the data source (such as a column header), while *Description* allows concise free-text context, and *Generated By* records the provenance about the tool, workflow step, or instrument process that produced the values. Where possible, values for Explication, Object Type, and Unit should be provided as *ontology terms* rather than free text. Referencing community ontologies disambiguates meanings, promotes interoperability across disciplines and software, and enables automated validation and aggregation.

Operationally, a Datamap is simply a collection of Data Contexts, one per fragment that merits explicit semantics. Since the structural properties of a Data Context are format-independent, the same Datamap schema can be reused across heterogeneous data and throughout diverse workflows without redesign. The result is fragment-level, machine-actionable metadata that supports reuse of common annotation patterns, facilitates automated processing and integration, and advances modularity, extensibility, and long-term sustainability.

### 3.2 Integration into ISA

The Datamap enables the precise identification and annotation of fragments within heterogeneous research data. However, fragment-level semantics only become fully actionable when they are embedded in rich experimental context. This includes information about the materials, protocol executions, parameters, and sampling design that produced the data. Such context is critical for Findability and Reusability, because it supports inter-dataset comparisons that respect differences in experimental conditions and provenance.

To provide this context, we integrate Data Fragment Selectors (DFS) with the Investigation–Study–Assay (ISA) framework [20]. ISA represents experimental workflows as a directed acyclic graph (DAG) in which material and data entities are transformed by protocol executions (“processes”) along a process sequence. Both entities and processes can be annotated with ontology terms (e.g., characteristics, factor values, parameter values), enabling machine-interpretable descriptions that carry domain knowledge.

**Figure 3:**
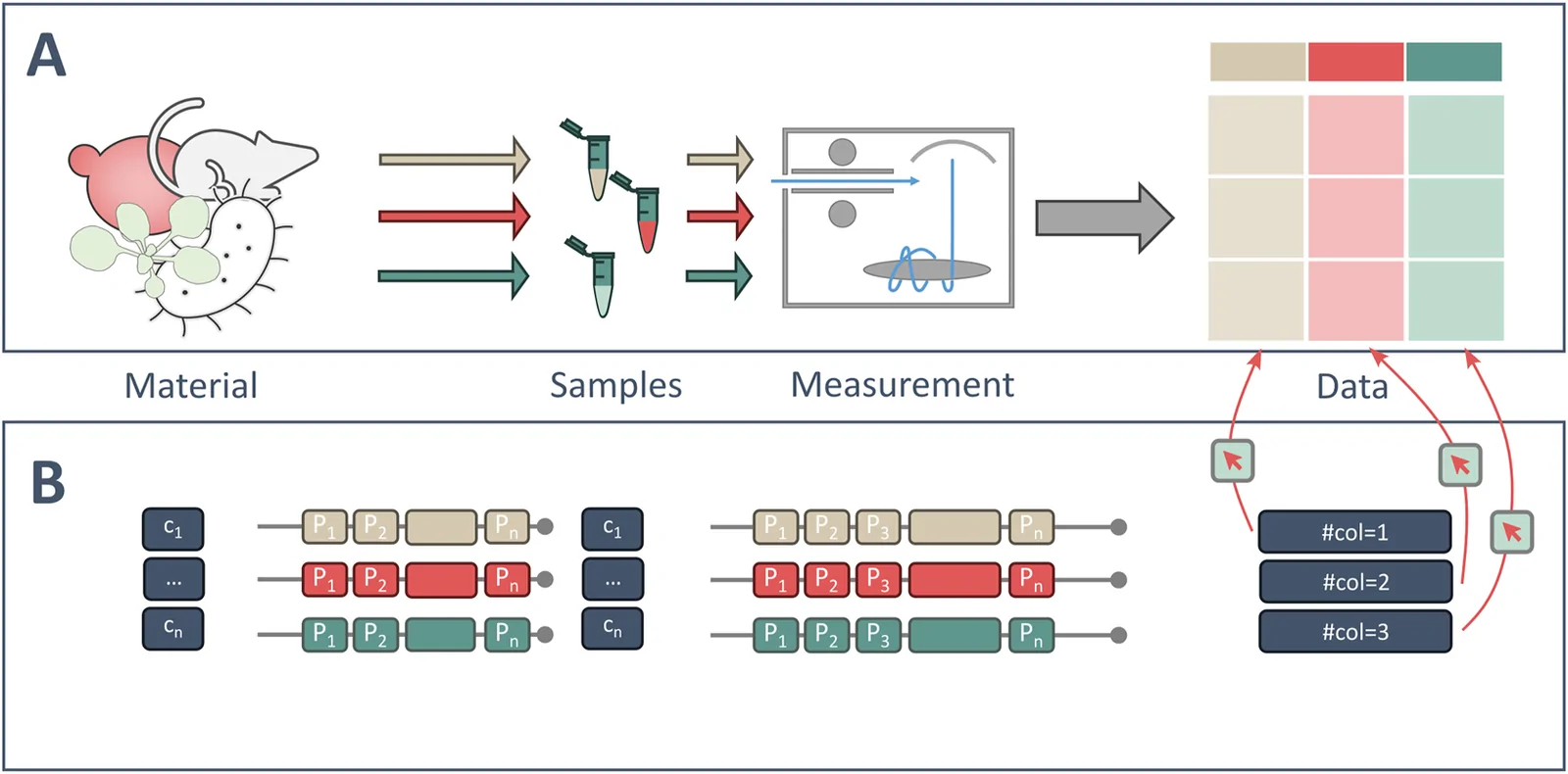
Data fragment selectors (DFS) seamlessly augment process-based metadata frameworks such as ISA. A, Schematic experimental workflow from material to samples and measurements, yielding a tabular data file in which each column corresponds to a sample-specific outcome. B, Process-sequence representation in ISA. With DFS, each column (or analogous fragment) is explicitly addressable and can be connected to its source material and protocol executions, thereby preserving fragment-level provenance and enabling semantic annotation.

Natively, ISA treats data files as atomic units. This defined level of granularity makes it difficult to record the provenance or meaning of sub-file entities (e.g., a row corresponding to one specimen, or a column representing a particular readout). We address this limitation by allowing ISA data entity references to carry, in addition to the file path or URI, an optional DFS that unambiguously addresses the fragment within that file. In practice, the DFS acts as a fragment identifier for the data node: edges in the ISA graph can then connect specific samples and protocol executions to specific within-file fragments, rather than to the whole file (illustrated in Figure 3). Importantly, this is a non-invasive extension. Existing ISA tooling that ignores the DFS continues to function (treating the reference as a conventional file), while DFS-aware tools can resolve and validate fragment-level semantics and provenance.

A key advantage of this design is that it remains file-format agnostic: neither Datamaps nor the ISA graph must parse the data file to understand the role of a fragment. The DFS provides the addressing scheme; ISA provides the provenance model. Together, they deliver a fine-grained, sample-aware metadata arrangement that is applicable across technologies and disciplines, facilitating robust reuse and cross-study analyses without imposing format-specific dependencies.

### 3.3 Representation in ISA-XLSX and RO-Crate

To operationalize fragment-level FAIRness we provide two interoperable serializations over a single abstract data model: *ISA-XLSX* within the ARC Scaffold and an ARC Datamap profile expressed as an RO-Crate. Both formats carry the same information; the mapping between them is designed to be lossless, enabling round-trips during validation and conversion.

We adopt ISA in spreadsheet workbooks because spreadsheets remain a widely-used medium for authoring experimental metadata, are easy to review in day-to-day lab practice, and impose a minimal training burden. Crucially, the community has already demonstrated that Excel workbooks can carry rich, ontology-backed annotations for life-science metadata entry. E.g., RightField embeds controlled vocabularies and ontology terms directly into Excel cells/columns via validation lists, showing the feasibility of a standards-compliant, spreadsheet-based curation workflow [30]. The Datamap tables we introduce deliberately follow the ISA annotation idiom (Data and Parameter columns). For ISA-literate users, this makes Datamaps immediately intuitive: authors continue to fill in familiar tables, while data columns can be extended in a low-barrier manner. In addition to the fragment selector itself, this includes a declared file format (media type) and a reference to the specification explaining this selector. These additions are sufficient to make fine-grained fragments first-class, machine-actionable objects without disrupting established spreadsheet practice.

As a packaging and exchange representation, we also provide a Datamap profile as an RO-Crate [31]. RO-Crate is a community standard for packaging research artifacts and their metadata as a portable, JSON-LD graph with explicit support for identifiers, provenance, and contextual relationships across heterogeneous data and software; its design and utility are formalized [23]. We selected RO-Crate as it offers a well-defined, file-system-friendly container, an extensible, profile-driven annotation model, and demonstrated machine-actionability for computational research. This machine-actionability has been evidenced by the Workflow Run RO-Crate profile that captures workflow execution provenance across systems [32]. Within our Datamap RO-Crate profile, we intentionally reuse Schema.org types and properties where possible (e.g., *Dataset*, *MediaObject*, *PropertyValue*, *DefinedTerm*) to maximize interoperability with the semantic web and with other RO-Crate profiles, while keeping our domain-specific additions to a minimum.

Because both serializations are views on the same abstract data model, we implement a one-to-one mapping between *ISA-XLSX* Datamap tables and Datamap RO-Crate entities/relations. As a result, the *XLSX* representation offers immediate usability for wet-lab scientists, while the RO-Crate representation yields a standards-compliant, semantically explicit research object suitable for automated validation, exchange, and long-term stewardship, without information loss between the two.

**Figure 4:**
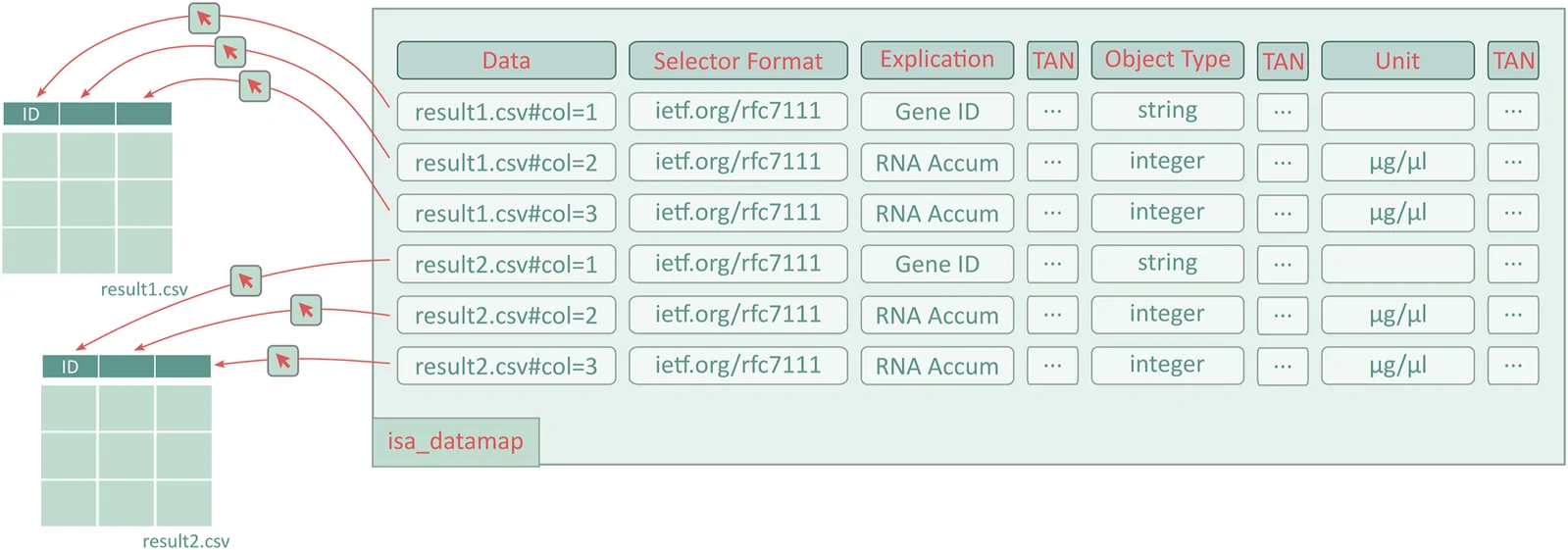
Tabular representation of the Datamap in the *isa.datamap.xlsx* file, specified in the *ISA-XLSX* specification. Each Data Context is represented by one row in the *isa_datamap* sheet, pointing to one segment of a data file. In this example, the Datamap is used to annotate two tabular data files, with each containing three columns. Note, that some columns have been completely left out and the Term Accession Number (TAN) column containing the URI to the ontology terms have been collapsed.

When compared, the Datamap XLSX (Figure 4) and the RO-Crate representation (Supplementary Figure S1) show close alignment. Both figures show annotation of the same underlying data, two tabular files. The RO-Crate representation is more hierarchical than the flat tabular annotation, with an explicit object existing for each data file and pointing to explicit objects existing for the file segments. The annotated values are identical, enabling a direct mapping based on fixed rules.

**Figure 5:**
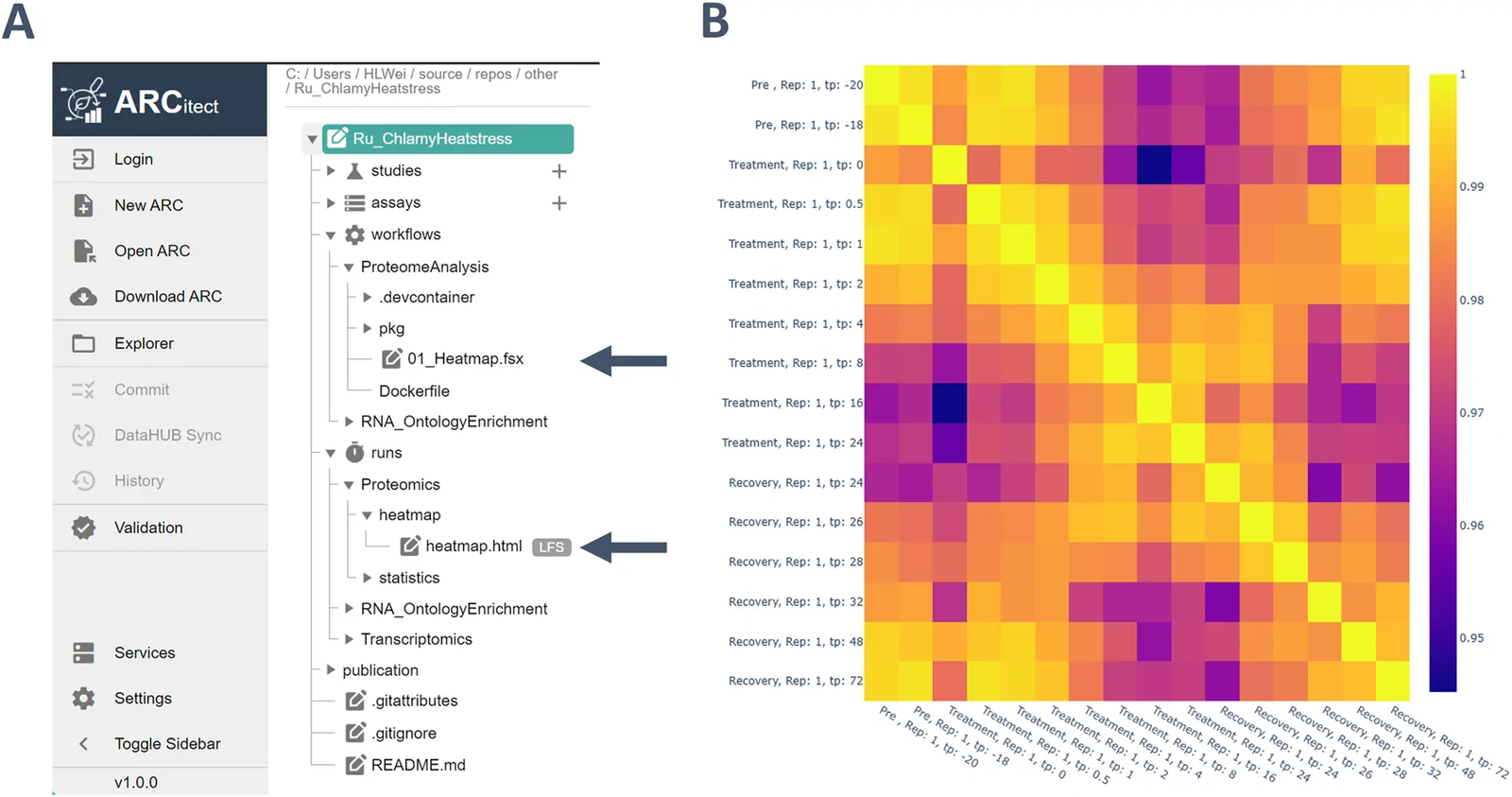
Real-world results of an explorative data analysis aided through DFSs and Datamap. *A* shows the ARC, visualized in the GUI-tool *ARCitect*, in which the data with its annotation is stored and the computation is defined and run. The position of the computation script and the result file have been marked with arrows. *B* shows the visual result of the data analysis.

In order to foster adoption of both the representations and the underlying concepts, the ARC tooling ecosystem catered especially towards users with no deep background in information technology now supports DFS and Datamap based data annotation. One central tool here is the ARCitect, which provides a clean graphical user interface (see Figure 5A) and is meant to enable researchers to thoroughly annotate their experiments [33].

### 3.4 Application

In typical journal publications, large datasets are deposited in external repositories and referenced in the data availability statement, whereas smaller datasets are often provided directly alongside the publication in a dedicated supplementary data section. The structure and format of such supplementary data strongly depend on how individual researchers aggregate and prepare them, as no standardized formats exist for maturely aggregated, processed datasets. Consequently, researchers usually shape their processed results into formats they believe are most comprehensible for data re-users.

To enhance comprehensibility, this process ideally includes concise annotations of the supplementary data. For example, clean, tabular datasets are commonly accompanied by an additional file or section that describes the contents of individual columns and rows as precisely as possible and links them back to the corresponding data processing steps from which the data originated. However, data annotation practices typically do not follow standardized routines or formats, which often leads to frustration for both data annotators and data reusers, particularly when locating and interpreting supplementary materials.

The Datamap not only allows substantial flexibility regarding the type, format, and structure of the datasets to be annotated, it simultaneously introduces a standardized structure and annotation workflow. Through the use of fragment selectors, Datamap enables dataset annotation at a highly granular level.

#### 3.4.1 Spreadsheet fragment selector

Data Fragment Selectors are intentionally designed to be extensible, allowing new format-specific selector definitions to be added, as annotation needs evolve. But we do not necessarily expect individual applied researchers to define their own specifications. Generic, domain-agnostic formats (like *CSV* or *JSON*) can be provided centrally and reused across disciplines, ensuring broad coverage for commonly used data representations. Widely used domain-specific formats (like *VCF* [34]), in turn, are expected to be created, curated, and maintained by domain experts such as data stewards or research data managers, thereby enabling consistent and reusable annotation practices within a given research community. For highly niche or inherently difficult-to-annotate formats, only limited structured annotation may be feasible at present. In the following, we showcase the addition of a generic, domain-agnostic fragment selector specification.

Spreadsheets, and in particular *XLSX* files, are a widely used format for storing biological research data [35]. The same annotation needs can be observed in ARC projects where results are curated in workbooks rather than *CSV* files. While RFC 7111 already covers fragment selection of *text/csv* files, there is no uniform way to cite substructures specific to spreadsheets [19].

Spreadsheets consist of multiple *sheets (or tabs)*, each containing a grid of *cells*. Users can also define named ranges and structured tables. Cell addresses usually use *A1 notation* with columns labeled alphabetically (*A*, *B*, …, *Z*, *AA*, *AB*, …) and rows labeled numerically. Cells are referenced by their column letter followed by their row number (e.g. *B3*). To close the annotation gap mentioned above, we propose a small, two-level selector syntax for spreadsheet formats [36]: a first-level *target* (sheet, table, or named) and a second-level *within-target* selector (range, cell, column, row), combined at most once via the “&” character:

**Figure:**
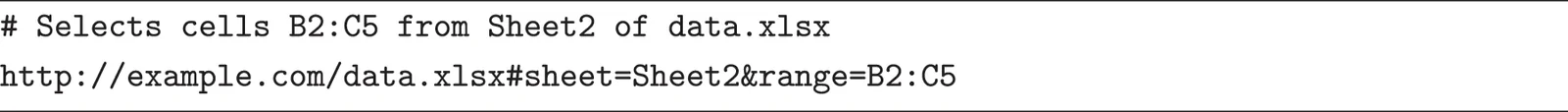


This simple selector format is the only mechanism needed to extend the Datamap model’s annotation capabilities for spreadsheet formats, as the annotation model itself is kept separate from the selector. Therefore, it also acts as a small example for the file-agnostic nature of the Datamap.

#### 3.4.2 Application in proteomics data analysis

The current state of the art in proteomics data analysis comprises a heterogeneous array of approaches. GUI-based tools such as Perseus are often used either standalone or in conjunction with script-based analyses [37]. Both approaches share that exploration, statistical analysis, and visualization rely on the incorporation of experimental metadata and tool specific metadata. Today, mapping metadata to datasets at the downstream end of the experimental pipeline is a labor-intensive, error-prone, and explicitly manual endeavor that revolves around looking up and mapping the metadata deemed relevant during analysis to identifiers of individual datasets (e.g., column headers in a CSV file). For the data analyst, this requires substantial knowledge of the experimental design and, frequently, guesswork about upstream metadata sources (e.g., lab notebooks), as well as manual review of tool documentation in search of explanations for individual columns.

In this example, we perform a exploratory quality control (QC) analysis that is routinely applied as an initial step in proteomics data analysis. We compute pairwise correlations between samples and examine replicate level patterns using a heatmap. Using this routine workflow as a running example, we illustrate how data analyses can follow more idiomatic programming routines, given the data being explicitly annotated using DFSs integrated into a process graph and into a Datamap. The concrete implementations are done in the context of an ARC and written in the functional programming language FSharp, but the programming patterns and the context in which the annotation patterns are applied are meant as generalizable procedures. As ARCtrl and ARCtrl.Querymodel are polyglot libraries, the functionality they provide is likewise made accessible to Python and JavaScript users via transpilation [38], 39].

##### 3.4.2.1 Dataset and code availability

The selected dataset represents the proteomics subset of a multi-omics study conducted by Zhang and colleagues [40]. Here, a time-course assessment of various biomolecules was performed to study the response of green algae to different levels of heat stress. This complex, metadata-rich experimental design is representative of modern, multidisciplinary biological studies and is ideally suited to demonstrate data-fragment selectors within the ARC framework. Both the code and the data shown are openly available at the PLANTdataHUB [41].

##### 3.4.2.2 Accessing the ARC

Using the *ARCtrl* library, the ARC under study can be used directly as the entry point for our exploratory analysis [38]:

**Figure:**
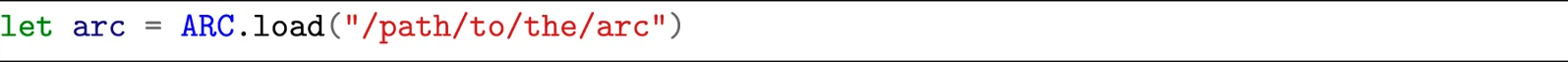


##### 3.4.2.3 Preparing metadata lookup

Since our goal is to compute replicate level statistics for a selected subset of the published dataset, we first define lookup functions that enable an unambiguous mapping from each downstream data entity to the metadata needed for interpretation of the QC plot (e.g., replicate group, condition, or time point). At this step, ontology-backed metadata annotation within the ARC framework enables the instantiation of ontological terms and the definition of lookup functions for retrieving relevant metadata from individual studies or assays [39]:

**Figure:**
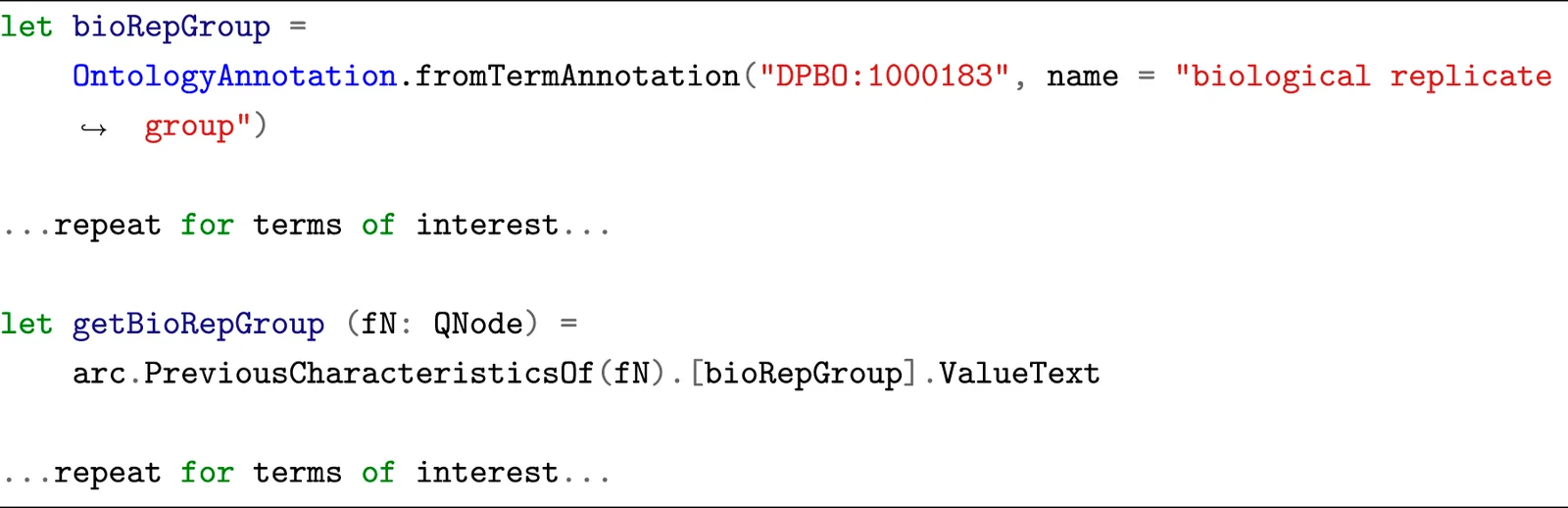


Crucially, these functions establish a reusable interface between the analysis code and the experimental design: instead of treating metadata as external context that must be manually reconstructed, the analysis can retrieve it directly from the ARC.

##### 3.4.2.4 Metadata-guided identification of samples and files to analyze

A typical first step in replicate QC is to select the subset of samples to be compared (e.g., one experimental condition and a defined replicate set) and then locate the corresponding quantitative proteomics matrix. To achieve this programmatically, the ARC can be queried for data nodes produced by the workflow and filtered using the accessible metadata. This enables inspection of available experimental groups and refinement of the selection to data nodes corresponding to a specific group and replicate number:

**Figure:**
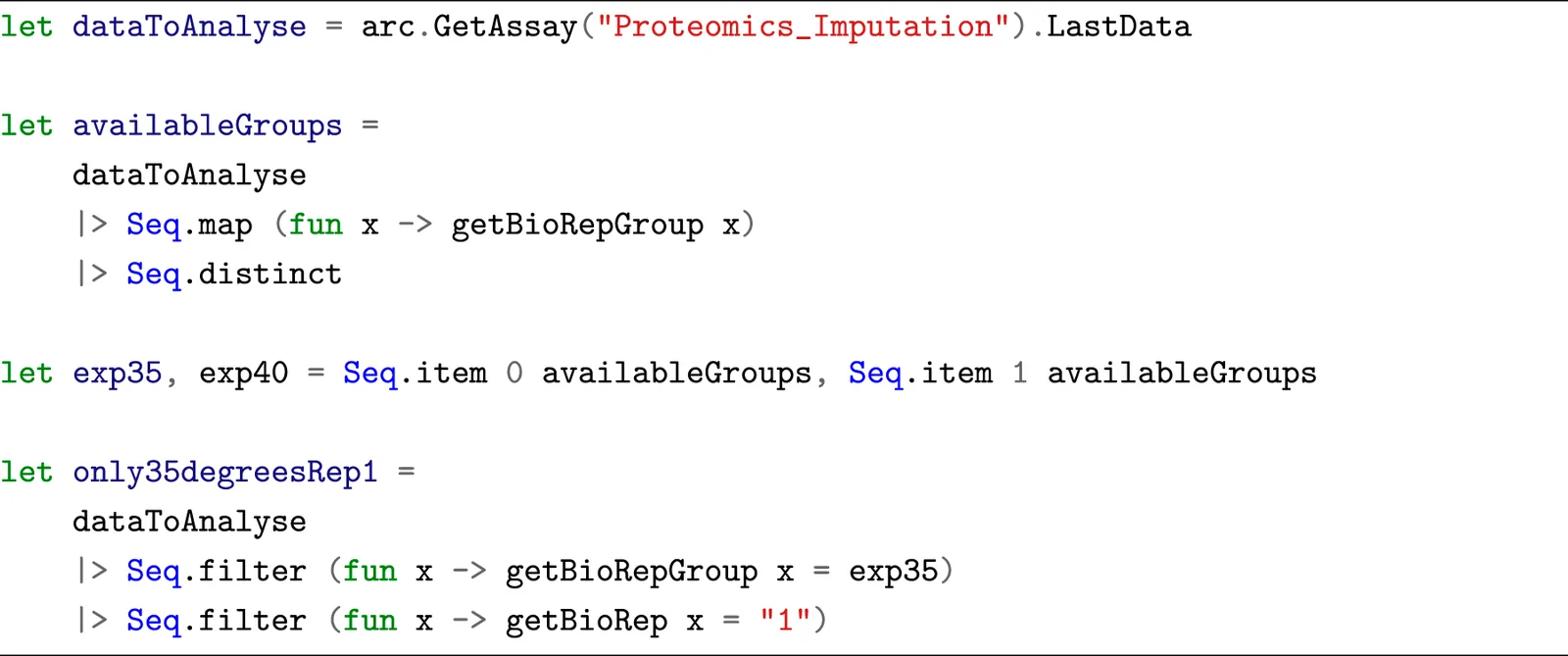


Because the data nodes are associated with Data Contexts, the available information can be used to infer file locations and formats and to read the corresponding data files with an appropriate software library:

**Figure:**
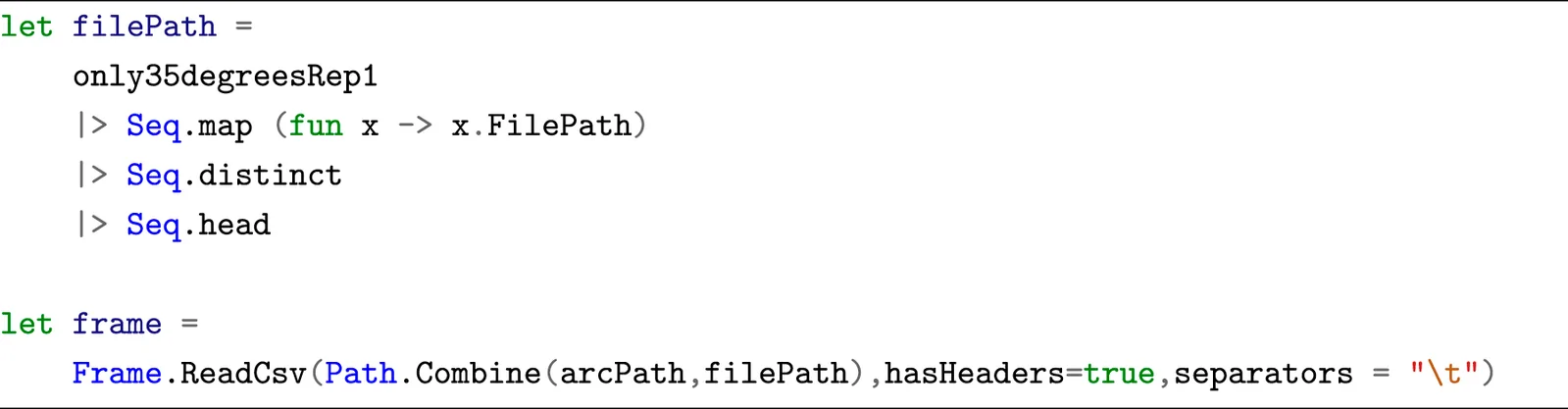


##### 3.4.2.5 Navigating data frames with data fragment selectors

Once the protein abundance matrix has been loaded, a routine QC correlation analysis typically involves (i) a indexing of proteins and (ii) the selection of the columns that correspond to the samples under comparison. In many quality-control workflows, this step requires manual interpretation of column headers and then mapping them back to the experimental context. In the ARC setting, given the file path, the corresponding data map can be queried to identify the Data Fragment Selector associated with the indexing column defined by the ontological term (*protID*). Using this selector, the appropriate column is referenced and the data frame is reindexed accordingly:

**Figure:**
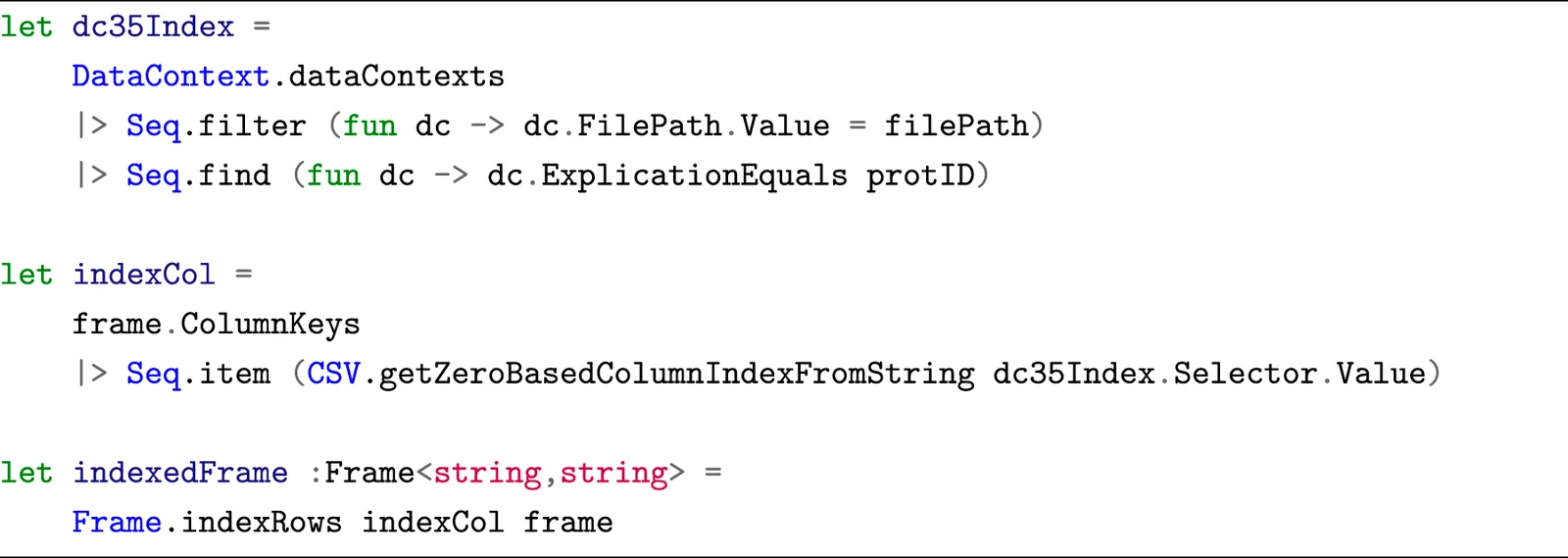


Similarly, DFSs of the data nodes can be leveraged to filter and retain only those data nodes corresponding to columns relevant to the analysis. Here, only protein abundance values are of interest and isolated by slicing the data frame while preserving metadata provenance:

**Figure:**
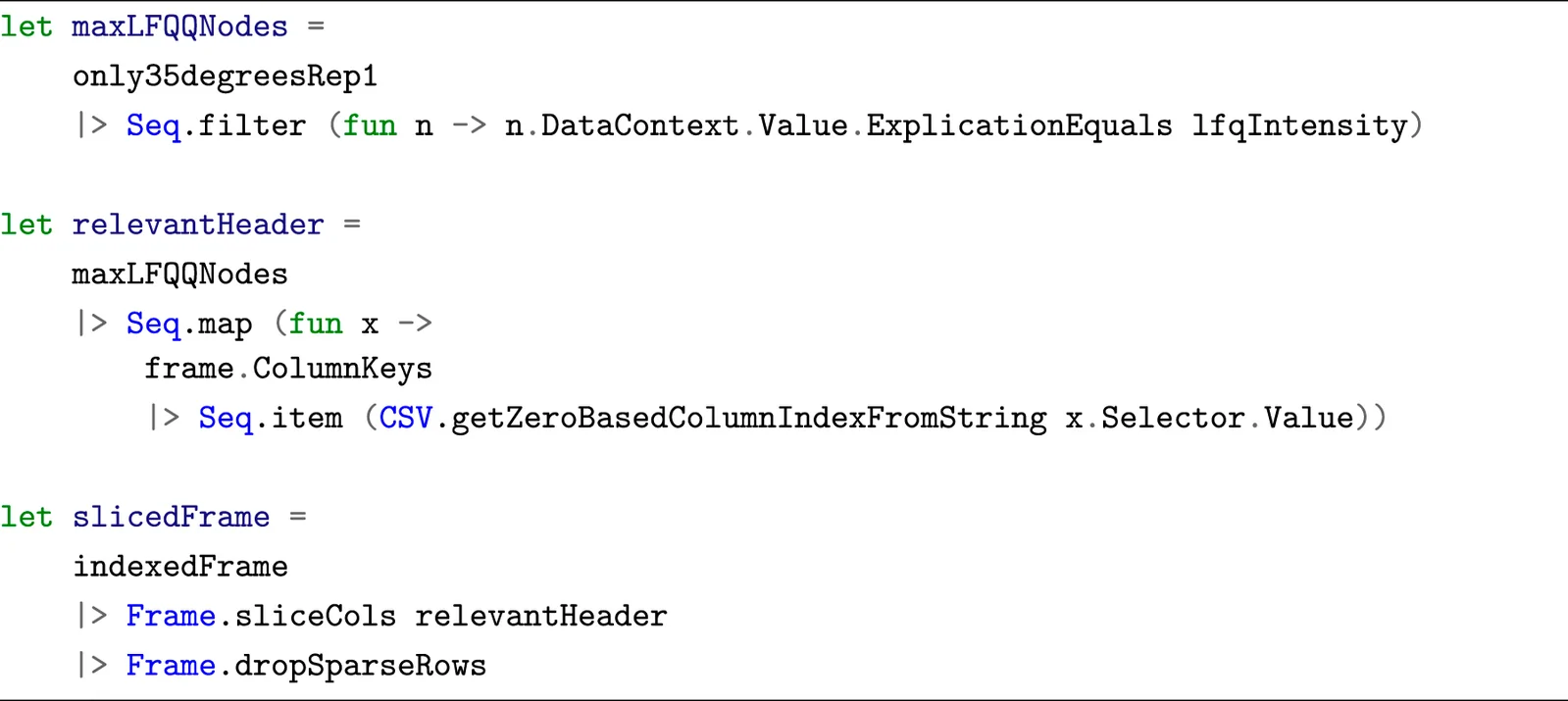


Additionally, a clear mapping can be created between ambiguously chosen data-frame headers and well-defined DFSs. This mapping is central for QC interpretation because it allows us to label samples in the heatmap using experimental metadata, rather than relying on file-internal naming conventions:

**Figure:**
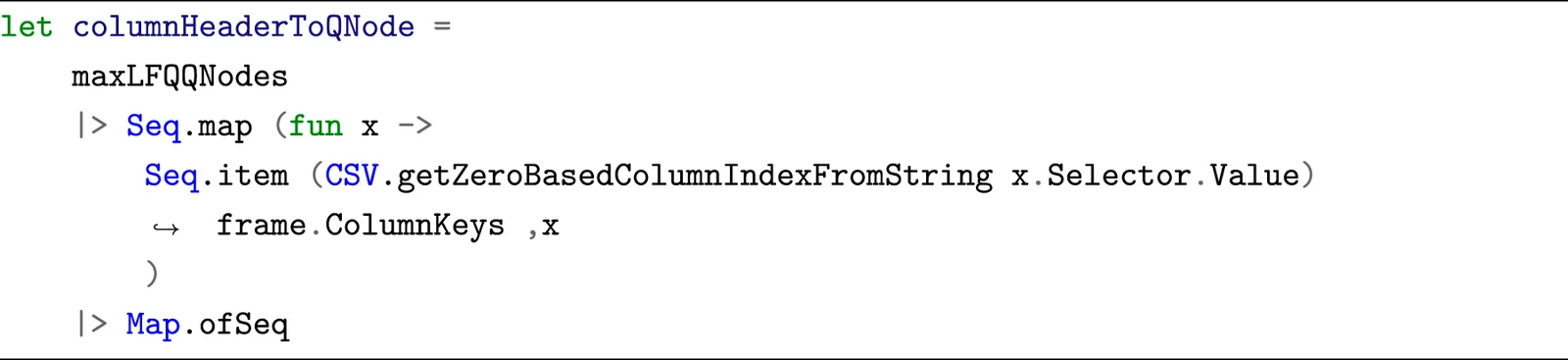


##### 3.4.2.6 Statistical assessment and visualization

With the relevant abundance matrix prepared, we can now perform the replicate QC itself. Pairwise correlations between samples provide a simple and widely used sanity check: biological replicates are expected to possess a relatively high within-group correlation, while unexpected patterns can indicate technical artifacts, sample swaps, or outlier measurements. Having prepared the frame, a correlation analysis can be performed and the resulting matrix visualized as a heatmap [42]. The established provenance enables styling of the plot using metadata retrieved directly from the ARC:

**Figure:**
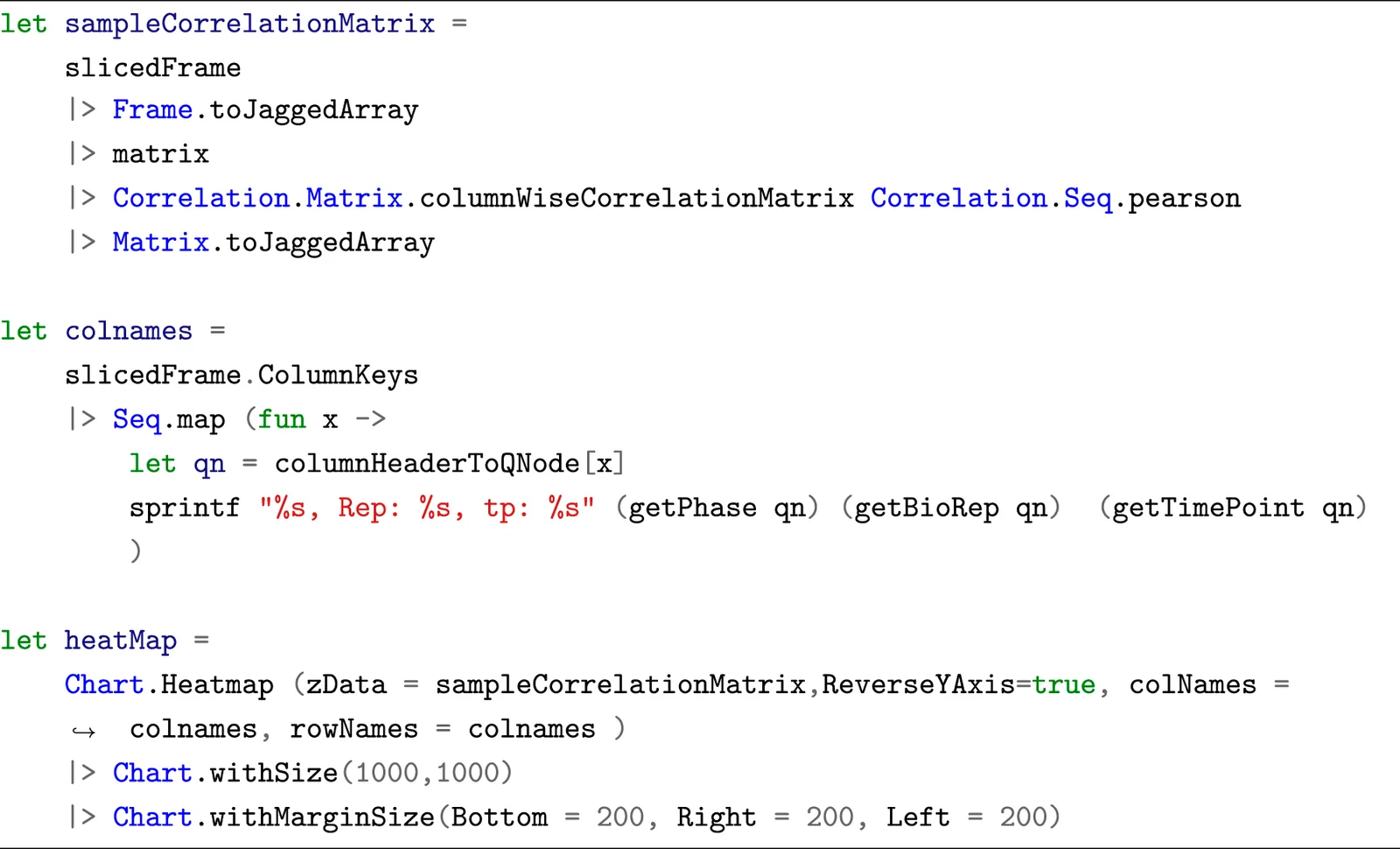


The result of this short, exploratory QC analysis can be observed in Figure 5. It shows both the state of the ARC visualized in the ARCitect tool (A), which in itself is a shareable result [41], and the final correlation heatmap (B). Importantly, the plot is annotated by querying metadata that are assigned to the data entities through DFSs, making the connection between the visualization and the experimental design explicit and reproducible.

## 4 Discussion

Associating data with both *structural* and *semantic* context remains a central objective in research data management and in operationalizing the FAIR principles [1], 43], 44]. However, frameworks that couple these layers too tightly by making annotation structure mirror idiosyncratic file layouts, tend to produce *data islands*. Biological data formats vary widely across domains and over time, raising the cost of interoperation across repositories and tool chains. In contrast, there is persistent value in comparing and reusing semantic content independently of concrete file encodings. We therefore argue for a principled *structural separation* of the two annotation components: keep structural description explicit and referenceable, while linking semantics via stable identifiers and vocabularies.

A key enabler of this decoupling is *fragment-level addressing*. By encoding structural information for described data in a fixed, predictable location of the annotation framework, we obtain a dependable scaffold for semantic annotation; at the same time, the structural layer *points to* an exchangeable, external resource (the actual file and its fragments), accommodating high variability in file formats without redefining the semantic layer. Building on principles defined in the Web Annotation Model, fragment selectors let us target precise regions of heterogeneous resources and bind them to semantic entities [27]. In our showcased real-world application of these principles, we could first query for the semantic entities of interest and then selectively activate file-structure–specific parsers only for the matched fragments, executing analysis according to rules declared in the data-file annotation.

Importantly, our approach is not an all-in-one parsing framework. Rather, it provides the explicitness required to faithfully represent real-world variability across instruments, formats, and laboratories. By making file structure and fragment selectors explicit and referenceable, we enable validation, linking, and reasoning to be handled by generic tools while leaving parsing to domain-appropriate libraries. In practice, this separation lets users integrate even *exotic* or more complex files without bespoke parsers, as demonstrated by our newly defined spreadsheet fragment selector [36]. This design is consistent with W3C best practices, which are prescriptive about outcomes (discoverability, interoperability, reusability) rather than technologies, and with FAIR’s emphasis on machine-actionability [1], 45].

We also find that integrating fragment selectors into ISA is natural in practice. ISA offers an articulate approach for annotating basically any real-world experiment through the use of ontology-enriched processes [20]. These processes track provenance of the experimental entities like samples and data files, but treat those files as atomic entities, losing track of provenance over more fine grained connections. DFSs can fill in this gap in a low friction way, even though we must remark, that canonical ISA tooling is currently not able to handle this for the core ISA serializations (ISA-Tab/ISA-JSON) [25].

Treating the *Datamap* as a first-class, provenance-agnostic entity has intrinsic value: it provides a stable schematization of data files that is independent of the workflow/provenance graph. This decoupling enables separate, non-conflicting layers for (i) structural properties (layout, addressing, typing) and (ii) biological/experimental semantics (traits, variables, units, methods). In contrast, a purely provenance-centric representation risks conflating context with structure. A prominent example can be seen by the mapping efforts of the MIAPPE community towards ISA compliancy [24]. The schematic separation of *observed variables* (trait/method/scale) from measurement tables has been a historic hurdle in fully representing MIAPPE in the ISA-Tab Format. Instead, maintaining this dictionary as a Datamap rather than embedding it in the process graph better reflects its role as a reusable semantic/structural reference. The core ISA model and tooling remain essential for experimental metadata, whereas the Datamap cleanly complements ISA by factoring out file schematization [20], 25].

Finally, we emphasize that the Datamap and our implementation of Data Fragment Selectors is not proposed as a final, closed solution. We advocate a general, standards-aligned approach that can co-evolve with its main representation container RO-Crate through community feedback and implementation experience, much like the Frictionless ecosystem. Our hopeful expectation is to chaperone the Datamap and its fragment selectors to mature as the community iterates on edge cases and contributes reference specifications and tooling [22], 23].

## Supplementary Material

Supplementary Material Details
